# Experimental Analysis of Critical Current and Alternating Current Losses of High-Temperature Superconductor Tape with Resin and Gallium-Indium-Tin

**DOI:** 10.3390/ma11040573

**Published:** 2018-04-08

**Authors:** Dongmin Yu, Yajie Sun, Huiming Zhang, Yuanzhu Meng, Huanan Liu

**Affiliations:** Department of Electrical Engineering, Northeast Electric Power University, Jilin 132012, China; d.yu@neepu.edu.cn (D.Y.); yjsun_neepu@foxmail.com (Y.S.); hz_neepu@foxmail.com (H.Z.); myz_neepu@foxmail.com (Y.M.)

**Keywords:** critical current, superconducting tape, liquid metal, resin, thermal contraction

## Abstract

This paper experimentally analyzes the critical current degradation and AC (alternating current) losses of second-generation (2G) high-temperature superconductor (HTS) tape during the impregnation process. Two impregnation materials were utilized: Gallium-Indium-Tin (GaInSn), and an epoxy resin, Araldite. The critical current of the impregnation materials was measured after different thermal cycles and compared with the tape with no impregnation process. The experimental results show that the critical current of Yttrium Barium Copper Oxide (YBCO) short samples varies between differently impregnated materials. The resin, Araldite, degraded the critical current; however, the GaInSn showed no degradation. Two degradation patterns with Araldite were identified due to the impregnation process, and the corresponding causes were analyzed. We further measured the AC losses of tapes impregnated with liquid metal at different frequencies, up to 600 Hz. Based on the experimental results, GaInSn liquid metal should be the most suitable impregnation material in terms of critical current degradation.

## 1. Introduction

Cryogenic coolers have been widely used in superconducting devices for their advantages in terms of easy maintenance and economical operations. The utilization of cryocoolers requires the impregnation of the superconducting tapes and coils to enhance heat transfer and thermal stability. Additionally, superconductors under high mechanical loads, such as high field magnets [1], generators [2], motors and transformers, should be stabilized by filling the voids in the structure. A lot of materials have been explored for both low-temperature superconducting and high-temperature superconducting coils.

Animal wax, such as beeswax, as well as petroleum-derived waxes, such as paraffin, were used in low-temperature magnets for a long time [3]. They were used as magnet coil filling agents to minimize the probability of thermal runaway events that can result from micro fracturing while impregnating the superconducting coils. However, these waxes have high thermal expansion and low thermal conductivity, and cracks emerge and develop quickly if the temperature is changed swiftly. Furthermore, due to their weak mechanical properties, the waxes are barely able to contribute to the overall mechanical stability of the magnets.

Recently, intensive research has been conducted into the application of epoxy resins, such as Stycast 1266, Stycast 2850FT and Araldite, as impregnation materials in high-temperature superconductor (HTS) tapes and coils. These resins are preferred for their high mechanical strength, good thermal conductivity and relatively small thermal expansivity. Barth tested the critical current of short REBCO samples with resin encapsulation [4]. Takematsu measured the critical current of YBCO coil with epoxy impregnation [5]. Their results revealed that the epoxies degraded the critical current of superconducting tapes and coils.

In order to avoid the degradation, various methods have been tried, including adding quartz powder to decrease the thermal expansion [4], depositing polyimide to wrap the tape [6], and winding the coil with tension [7]. Some other methods have also been proposed to avoid degradation. Zhang used a heat-shrink tube to insulate the HTS tape to prevent the delamination due to epoxy impregnation [8]. Her experimental results showed that the insulated coil had no current degradation before and after the epoxy impregnation process.

So far, although the degradation from epoxy resin impregnation has been discussed, few papers have systematically implemented experiments to study the degradation. Furthermore, the influence of impregnation on AC loss has rarely been reported. This paper aims to experimentally analyze the influence of the impregnation process on the critical current and AC loss of short YBCO tapes. In Section 2, the experimental setup for *I*_c_ measurement is presented with different impregnation materials. Section 3 presents the critical current and AC loss measurements and discusses various factors that influence the critical current. The findings of this investigation are summarized in Section 4. 

The contribution of this paper can be summarized as follows: (1) The liquid metal, GaInSn, is applied to impregnate the YBCO tapes for the first time; then, the critical current and AC losses are measured. Our experiments reveal that the usage of GaInSn doesn’t degrade the critical current, and two possible reasons are identified; (2) This paper experimentally investigates the E-I curves of YBCO tape impregnated by Araldite and two patterns—a resistive pattern and a skyrocketing pattern—are observed for the first time; (3) We further explore the skyrocketing pattern by inserting direct current (DC) with a different ramp rate and find that the skyrocketing pattern shows a ramp rate dependency, which is due to thermal phenomenon. 

## 2. Experimental Setup 

**Figure 1:**
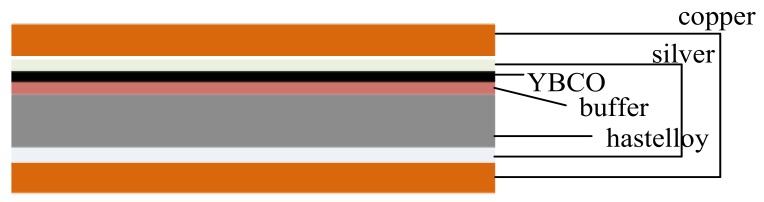
Typical configuration of 2G HTS tape.

YBCO tapes supplied by SuperPower (Schenectady, NY, USA) [9] and SuperOX (Moscow, Russia) [10] are tested. They are all 4 mm wide tapes with stabilizer layers made of copper on both sides. The typical structure of the superconductors is shown in Figure 1. The black region stands for the superconducting YBCO layer.

Short pieces of tape 20 cm in length are stripped off to measure the self-field critical current before impregnation in liquid nitrogen. Two materials are used to impregnate the 2G HTS conductor. One is a liquid metal, named Gallinstan (Geschwenda, Germany), which consists of three major elements: Gallium, Indium and Tin. It is a metal with a melting temperature of −19 °C and is liquid at room temperature. Gallinstan is non-toxic and has very good thermal conductivity [11]. Therefore, Gallinstan is a potential material for impregnation. Araldite epoxy resin (Denver, CO, USA) is prepared according to Table 1, which shows the mixture ratio and cure conditions.

**Figure 2:**
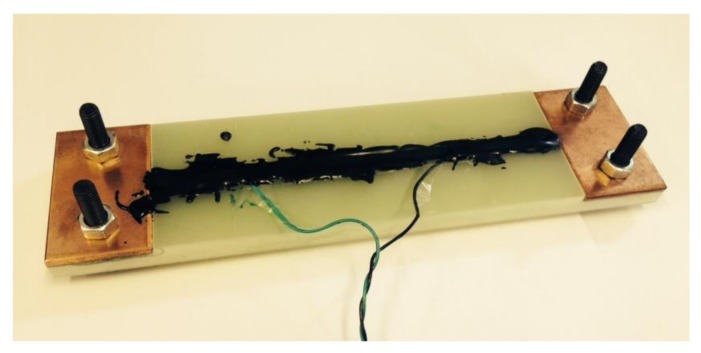
Prepared sample with Stycast black impregnation.

All samples are prepared using identical methods. Voltage taps are soldered onto the upper surface of the tape with 10 cm gaps in between. The critical current is measured in a liquid nitrogen bath (77 K) in self-field condition. The critical current of the short tape is defined by a critical electrical field *E*_c_ of 1 µV/cm. The tapes are first cleaned with Acetone. Then they are encapsulated into a U-shape former made of glass-fiber-reinforced-plastics (G10) using a glue or resin mixture, as shown in Figure 2. Two pieces of copper plates are fixed to the holder at both ends, and these are connected to the current lead. The glue or resin mixture is distributed homogeneously by curing the impregnation.

The transport current is provided by an HP 6681A DC power supply (Palo Alto, CA, USA). The maximum output voltage is 8 V and the maximum current 580 A. During the critical current tests, the power supply operates in linearly varying current mode. The amplitude and the duration of the current output are controlled either remotely by the standard commands for the programmable instruments over an IEEE 4888 bus or locally by an analog button.

**Figure 3:**
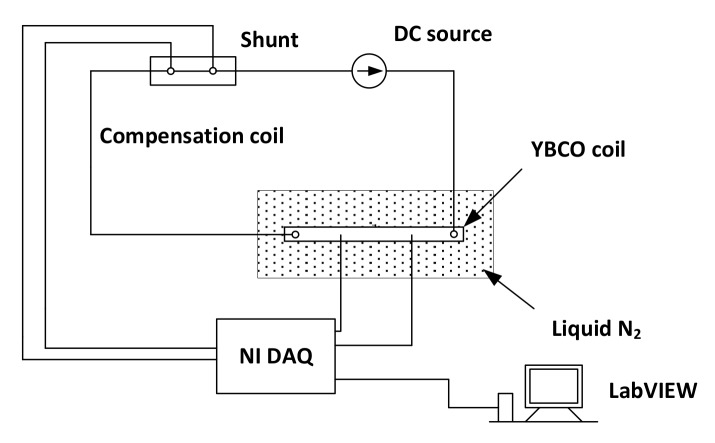
Critical current measurement circuit.

The Labview Version 7.0 program (National Instruments, Austin, TX, USA) is designed to measure the critical current of short tape samples, and this program is able to adjust the voltage rate. It consists of measuring the voltages of the taps on the samples and the currents flowing in the tapes, as shown in Figure 3. Then, the averaged electric field between the voltage taps, denoted as E, is calculated by dividing the voltage by the gap length, which is 10 cm in our experiments. The real-time electric field-current (E-I) curves are drawing during the experimental process. These curves are fitted by the following equation:

(1) $$E=E_{c}{\left(\frac{I}{I_{c}}\right)}^{n}\Theta \left(I-I_{c}\right)$$

where *E*_c_ is the critical electric field and its value is 1 µV/cm, *I*_c_ is the measured critical current with the criteria of 1 µV/cm, n value is the fitted exponent parameter, Θ(x) is the Heaviside step function.

The SCXI-1314 is an 8-channel voltage card allowing any device that alters its output voltage in response to change in the physical condition that is being measured [12]. The card can be linked to LabVIEW and programmed to acquire or generate voltage signals. A temperature sensor is used to monitor the working temperature of the superconductor in liquid nitrogen. Balancing the accuracy, operation and cost, a thermocouple PT100 is chosen. Each measurement is repeated for three samples.

**Figure 4:**
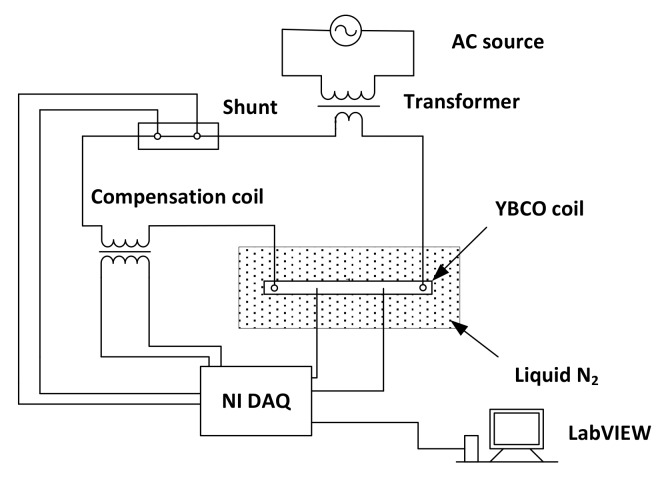
AC loss measurement circuit.

The AC losses are measured after the *I*_c_ characterization [13]. Figure 4 shows the circuit diagram of the electrical transport AC loss measurement by the electrical circuit with the NI (National Instruments) data acquisition system (DAQ). The sample is supplied with an alternating current (AC) using a power amplifier controlled by a signal generator. The voltage loss signal between the terminals of the tape is superposed with the component signal, which can be eliminated using a cancellation coil. The voltage signal after compensation is amplified and filtered to remove the harmonic components using a high-accuracy DAQ measurement system in the LabVIEW program. The transport AC loss of the tape per cycle is given as:

(2) $$Q=I_{\mathrm{rms}}V_{\mathrm{rms}}/f$$

where f is frequency, $I_{\mathrm{rms}}$ and $V_{\mathrm{rms}}$ are the root-mean-square current and voltage within the tape, respectively. $V_{\mathrm{rms}}$ is the voltage after compensation, as shown in Figure 4.

## 3. Results and Discussion 

### 3.1. Critical Current

**Figure 5:**
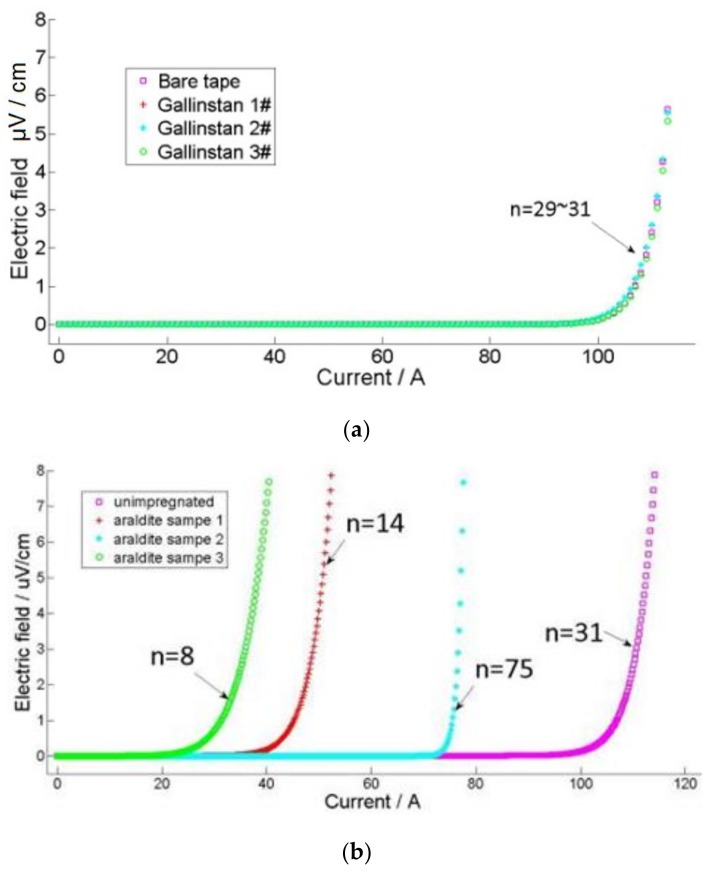
E-I curves with different impregnation materials. (**a**) Gallinstan; (**b**) Araldite.

Impregnation materials have different *I*_c_ and n value degradation effects on SuperPower tapes. Figure 5a shows the E-I curves of samples with Gallinstan. As shown in Table 2, the critical currents and n values are above 100 A and are almost the same as the unimpregnated samples, which means Gallinstan is barely able to degrade the YBCO tapes. There may be two possible reasons for this. The first reason is that the thermal mismatch is not big enough to induce cracks on the YBCO layer [14]. The second reason may be that the wettability of Gallinstan on copper is not very good [15]. This would reduce the bonding between the Gallinstan and the tape, thus reducing the tension in the tapes.

Unlike Gallinstan, resin shows degradation at different levels. For Araldite, the samples degrade to critical currents of 30 A, 45 A and 78 A. At the same time, the n values with Araldite are 75, 14 and 8. This means that Araldite substantially degrades the critical currents and change the n values. This degradation is due to the thermal shrinkage coefficients. According to the literature, the thermal contraction of Araldite from room temperature to 77 K is 1.2% [16]. However, the thermal shrinkage for the YBCO stabilizer, copper, is about 0.26%. Thus, the thermal mismatch between the resins and stabilizer will induce strain within the superconductor tape, and finally degrade the *I*_c_. This degradation is related to the degree of thermal mismatch. In order to enhance the critical currents for power device and magnetic applications, other measurements, such as with the addition of micrometer-level powders, are necessary in order to improve the contraction during the cooling process. 

**Figure 6:**
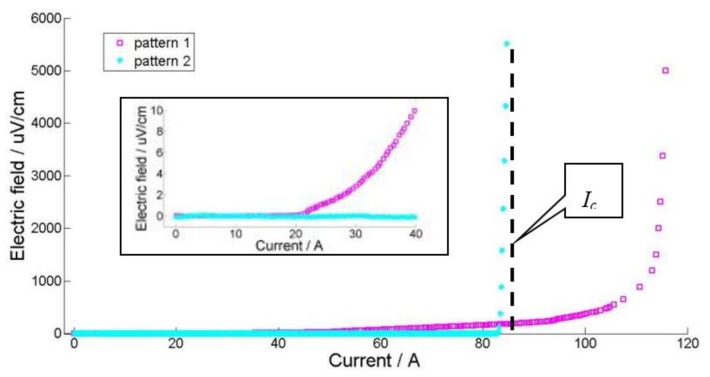
Two type of degradation patterns in E-I curve, pattern 1 is referred to as the resistive pattern, pattern 2 is named the skyrocketing pattern. The zoomed picture shows the transition of pattern 1 from the original point.

In our experiments, two E-I curve patterns are observed, as shown in Figure 6. The first is pattern 1 in the graph. This pattern features in the resistivity transformation during the ramping up process. The tape shows resistivity when the tape ramps to 20 A; the voltage goes up continuously as the current increases, until the current approaches the critical current, represented by the vertical dashed line. We call this the resistive pattern.

The second pattern shown in Figure 6 has a very rapid superconducting transition. Before the tape quenches, the voltage rockets up at 84 A. This transition happens within 0.1 s, with the electric field increasing from 0 to several thousands of µV/cm. We call this the skyrocketing pattern. 

**Figure 7:**
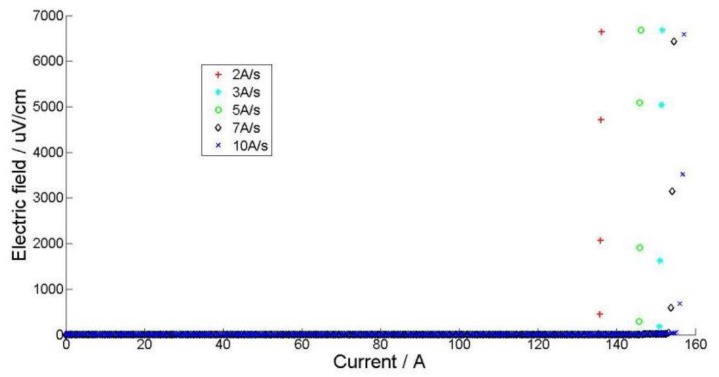
SuperOX tape with different ramp rates.

In order to investigate pattern 2 further, different ramp rates were attempted in Figure 7. The critical currents vary with different current ramping rates for the same sample. As we can see, as the ramp rate increases, the rocketing-up of the current increases considerably. When the ramp rate is 2 A/s, the current is about 138 A; however, this current increases to 158 A at 10 A/s. 

Based on this ramp rate dependency, we infer that this transition is related to the thermal phenomenon. When the current reaches the transition current, the sample quenches quickly and the temperature quickly rises beyond the critical temperature, so the sample shows swift transition. The quicker the ramp rate, the slower the heat generated by the disturbance, the higher the transition current. Similar results have been reported by Yanagisawa [6].

### 3.2. AC loss

**Figure 8:**
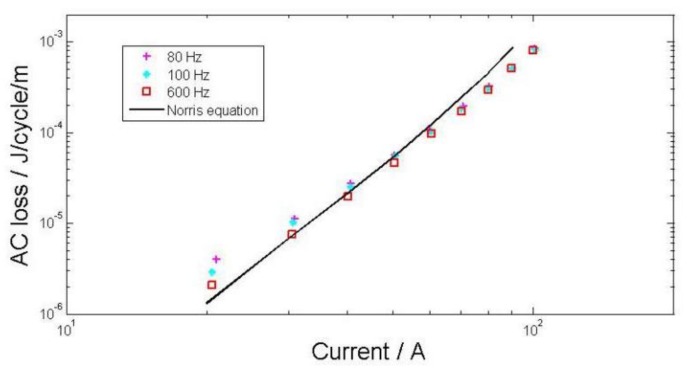
AC losses with non-insulated tape compared with the Norris equation.

The results of transport current loss measurements in self field are presented for unit length by dividing the distance between the voltage tapes. The measured values are compared to predictions from the Norris equation for an infinite slab [17]. This equation calculates the loss in isolated wires of rectangular cross section. The AC losses measured in a single piece of bare tape are shown as a function of applied current in Figure 8. Each series of symbols with a different style correspond to a different frequency. The tapes have critical currents of 110 A in self field. 

**Figure 9:**
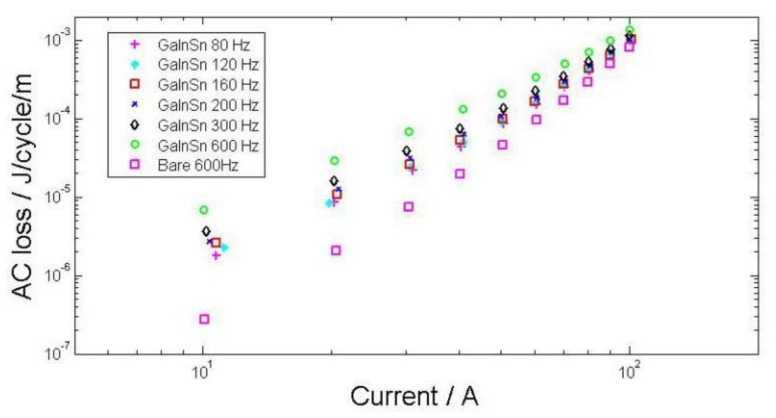
AC losses with Gallinstan impregnated tapes compared with non-insulted tape.

AC loss measurement results obtained using Gallinstan impregnation are displayed as symbols in Figure 9. They are compared to bare tape with the same parameters as shown in Figure 8. For impregnated tapes, there are clear differences with the different applied frequencies, from 80 Hz to 600 Hz. The measured AC losses increase non-linearly with the applied current frequencies. This increase is explained by the induced eddy current in Gallinstan.

## 4. Conclusions

This paper experimentally compares the influence of different impregnation materials on coated conductors. These impregnation materials include liquid metal, Gallinstan, and the epoxy resin, Araldite. The experimental results show that Gallinstan is hardly able to degrade the tape, while resins result in obvious degradation. The degradation is closely related to the thermal expansion mismatch. For tapes impregnated by Araldite, two degraded E-I curve patterns are observed, one behaves like a resistive transition, the other is a skyrocketing transition. For the skyrocketing pattern, when the current reaches the transition current, the sample quenches quickly and the temperature quickly rises beyond the critical temperature, so the sample shows a rapid transition. Finally, the AC losses are discussed after impregnation. The Gallinstan impregnation experiments show that AC losses have frequency dependency, and this is due to the eddy loss within the Gallinstan.

## Figures and Tables

**Table 1:** Resin ratio and cure condition.

Resin Component		Ratio in Weight	Cure Condition
Araldite	Araldite DBF	100	48 h, 25 °C
	Hardener HY951	10	

**Table 2:** Critical current and n values with different impregnation materials.

Material	Gallinstan		Araldite	
	I_c_	n	I_c_	n
Sample 1	106.2	29	45	14
Sample 2	106.7	31	80	75
Sample 3	106.4	31	32	8
